# Supplementary material for: Epigenetic editing of Cartpt promotes acquisition and extinction of cocaine memory
Source: bioRxiv. 2025 Nov 20:2025.11.20.689329. Preprint. [Version 1] doi: 10.1101/2025.11.20.689329 (PMC12667977; doi:10.1101/2025.11.20.689329)
Supplement: Supplement 1 [file media-1.pdf]

Extended Data for

Epigenetic editing of Cartpt promotes acquisition and extinction of cocaine memory.

Julia J. Winter[1,2], Marisol Hooks[1,2], Keegan S. Krick[1,2], Alexandra Goldhamer[1,2], Ronald W. DiTullio[3], Kyle S. Czarnecki[1,2], Claude-Ericka Ekobeni[1,2], Chloe Han[1,2], Kiara L. Rodríguez-Acevedo[1,2], Brandon W. Hughes[4], Molly Estill[4], Collin D. Teague[4], Aarthi Ramakrishnan[4], Li Shen[4], Eric J. Nestler[4], Elizabeth A. Heller*[1,2]

Affiliations:

[1] Epigenetics Institute at the Perelman School of Medicine at the University of Pennsylvania, Philadelphia, PA, 19104, USA

[2] Department of Systems Pharmacology and Translational Therapeutics, Perelman School of Medicine, The University of Pennsylvania, Philadelphia, PA, 19104, USA

[3] Institut de la Vision, Sorbonne University, 75012 Paris, France

[4] Nash Family Department of Neuroscience and the Friedman Brain Institute, Icahn School of Medicine at Mount Sinai, New York, NY, 10029, USA

*Corresponding author

Elizabeth A. Heller, PhD

10-115 Smilow Center for Translational Research

3400 Civic Center Boulevard, Building 421

Philadelphia, PA 19104-5158

Email: eheller@pennmedicine.upenn.edu

Supplementary Figure S2.

a CRISPR plasmid maps

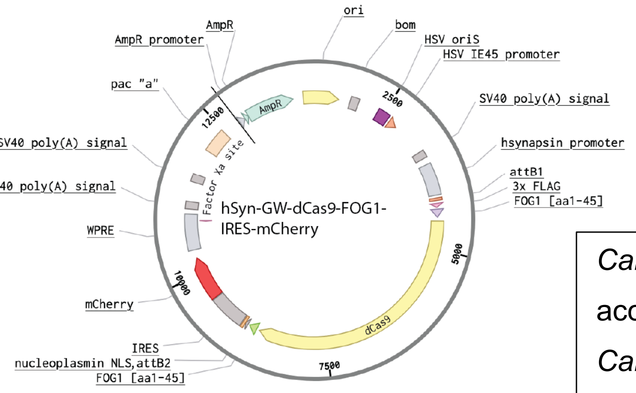

Cartpt-sgRNA sequence TSS -205:

accggacgtggtaccagtcc

Cartpt-sgRNA sequence TSS -526:

tattgtggcgagggtaacta

control-sgRNA:

cgaggtattcggctccgcg

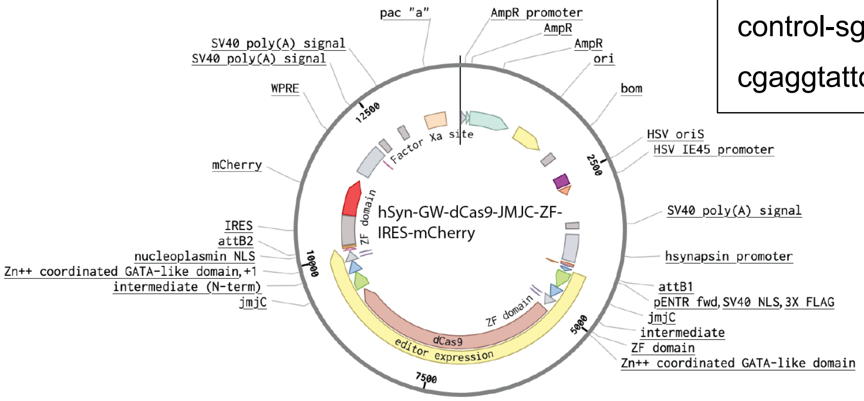

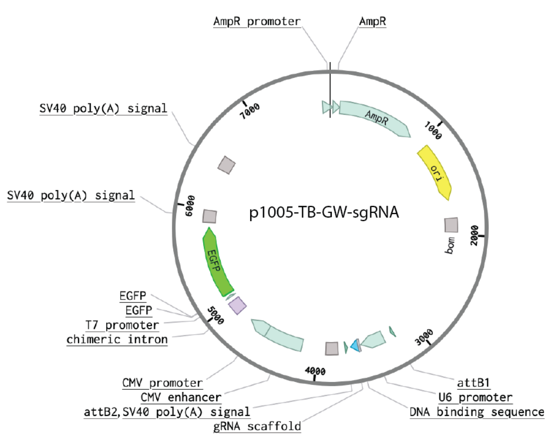

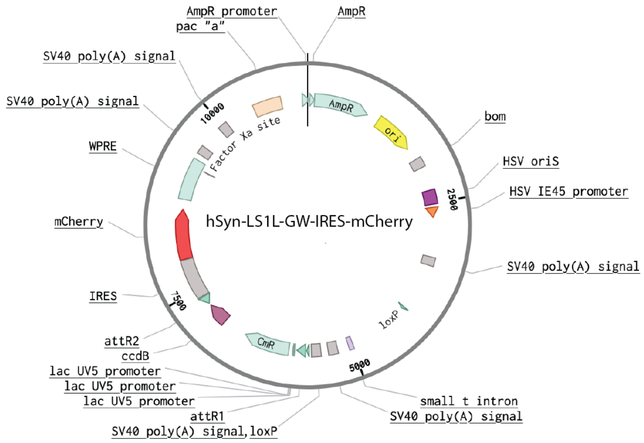

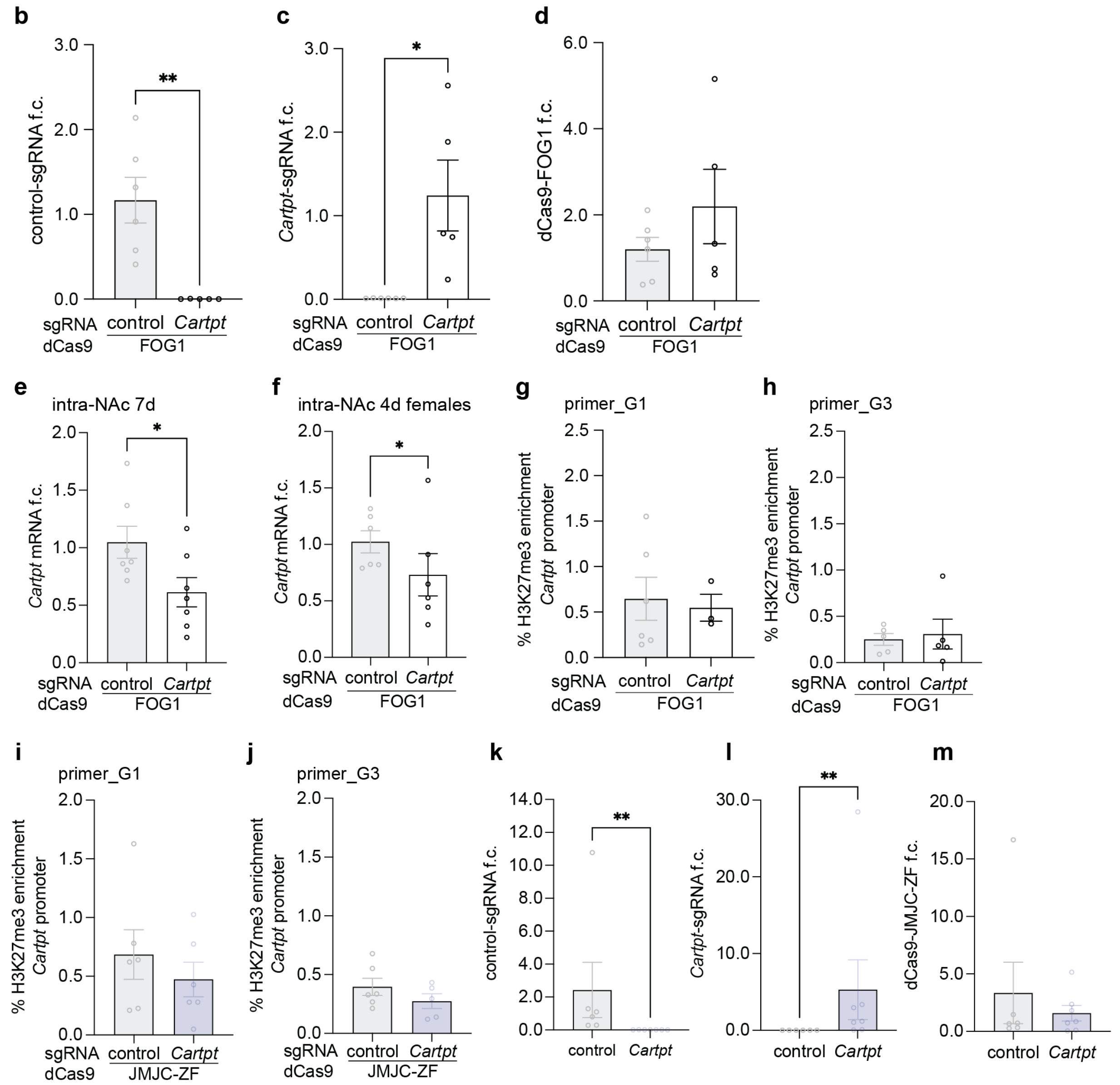

Figure S2. Control qPCRs of epigenetic editing and additional qChIP analysis.

a Plasmid maps of all constructs used in this study, generated with Benchling.com. Single-guide RNA sequences are as followed: *Cartpt*-sgRNA sequence TSS -205 accggacgtggtaccagtcc; *Cartpt*-sgRNA sequence TSS -526 tattgtggcgagggtaacta; control sg-RNA sequence cgaggtattcggctccgcg. **b** Control qPCR analysis for correct plasmid expression confirmed detectability of control-sgRNA in control samples with dCas9-FOG1, two-tailed t test, Mann-Whitney U=0, **p=0.0043, n=5-6. **c** *Cartpt*-sgRNA was only detectable in targeting samples and dCas9-FOG1 via qPCR, unpaired two-tailed t test with Welch's correction *p=0.0442, t=2.897, df=4.000, n=5-6. **d** Control qPCR confirmed equal dCas9-FOG1 expression in control and *Cartpt*-

sgRNA samples, unpaired two-tailed t test with Welch's correction p=0.3245, t=1.097, df=4.823, n=5-6. **e** *Cartpt* mRNA remained downregulated in NAc at day 7 after injections, compared to control-sgRNA, unpaired two-tailed t-test with Welch's correction, *p=0.0398, t=2.308, df=11.91, n=7. **f** *Cartpt* mRNA was downregulated in adult female mice treated with *Cartpt*-sgRNA and dCas9-FOG1 on experimental day 4 compared to control-sgRNA, paired one-tailed t test *p=0.0453, t=2.092, df=5, n=6. **g** Enrichment of H3K27me3 at the *Cartpt* promoter by dCas9-FOG1/*Cartpt*-sgRNA was not detectable when assessed by primer_G1, paired t test, p=0.4576, t=0.9131, df=2, n=3-6, relative to dCas9-FOG1 and control-sgRNA. **h** Enrichment of H3K27me3 at the *Cartpt* promoter by dCas9-FOG1/*Cartpt*-sgRNA was not detectable when assessed by primer_G3, Wilcoxon matched-pairs signed rank test, two tailed, p>0.9999, n=5, relative to dCas9-FOG1/control-sgRNA. **i** De-enrichment of H3K27me3 at the *Cartpt* promoter by dCas9-JMJC-ZF/*Cartpt*-sgRNA was not detectable when assessed by primer_G1, unpaired t test with Welch's correction, p=0.2164, t=0.8213, df=8.937, n=6, relative to dCas9-JMJC-ZF/control-sgRNA. **j** De-enrichment of H3K27me3 at the *Cartpt* promoter by dCas9-JMJC-ZF/*Cartpt*-sgRNA was not detectable when assessed by primer_G3, unpaired t test with Welch's correction, p=0.1207, t=1.254, df=8.988, n=5-6, relative to dCas9-JMJC-ZF/control-sgRNA. **k** Control qPCR analysis for correct plasmid expression confirmed detectability of control-sgRNA in control samples with dCas9-JMJC-ZF, two-tailed t test, Mann-Whitney U=0, **p=0.0012, n=6-7. **l** *Cartpt*-sgRNA was only detectable in targeting samples and dCas9-JMJC-ZF via qPCR, two tailed t test, Mann-Whitney U=0, **p=0.0012, n=6-7. **m** Control qPCR confirmed equal dCas9-JMJC-ZF expression in control and *Cartpt*-sgRNA samples, two tailed t test, Mann-Whitney U=19, p=0.8357, n=6-7.

Supplementary Figure S3.

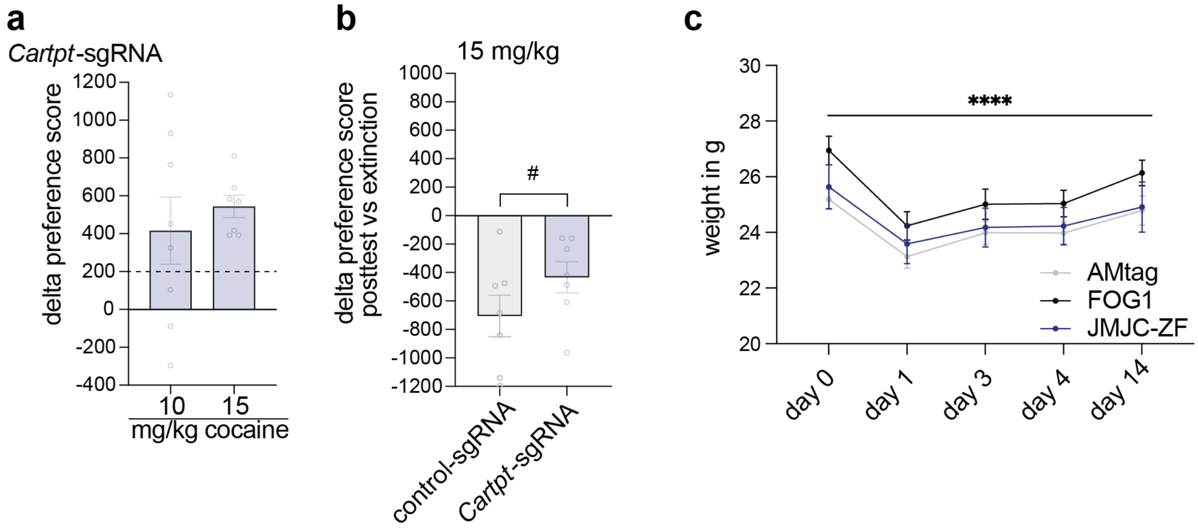

Figure S3. Additional cocaine CPP data after epigenetic editing with dCas9-JMJC-ZF.

a Dose response curve of cocaine CPP at 2 different doses, 10, and 15 mg/kg cocaine. *Cartpt*-sgRNA: epigenetic editing with dCas9-JMJC-ZF at *Cartpt* narrows cocaine preference at 15 mg/kg compared to 10 mg/kg, unpaired t test with Welch's correction, p=0.2552, one-tailed, t=0.6871, df=8.510. **b** Analysis of delta preference score posttest vs extinction of CPP at 15 mg/kg cocaine. Extinction of mice treated with dCas9-JMJC-ZF and *Cartpt*-sgRNA was decreased compared to control-sgRNA, unpaired t test with Welch's correction, #p=0.0821, one-tailed, t=1.489, df=11.12. **c** Tracking of body weight over 14 experimental days, two-way ANOVA, significant effect of time ****p<0.0001 and animal p<0.0001, no effect of treatment p=0.2727, n=8.

Supplementary Table S1. Primer design.

Primer	Sequence
*Cartpt*_ori	fwd: TACTCTGCCGTGGATGATGCGT<br>rev: TCGGAATGCGTTTACTCTTGAGC
*Cartpt*_MDC (Carpenter et al., 2020)	fwd: ACGAGAAGGAGCTGATCGAA<br>rev: TCTCTGAGGGGAACGCAAAC
G1_primer	fwd: GGGTGTAGCAGCAAGAAGGA<br>rev: GTACCACGTCCGGTTCTCTC
G2_primer	fwd: CTGAACGGGAGCGAGAGAAC<br>rev: TGTGTACACGAGTGCAGGTG
G3_primer (Carpenter et al., 2020)	fwd: ACACAAGAGCCGTCAATTCCA<br>rev: TCGAGTTCCCAACACCGC
pENTR	fwd: CACCATGCCCAAGAAGAAGAGGAAG<br>rev: CTTTCATGCGGCCGCAGGTC
dCas9-FOG1	fwd: CAGAGAGGAGGTGCAGTTGG<br>rev: CCGTGCTGTTCTTTTGAGCC
dCas9-JMJC-ZF	fwd: ACGGTGGTAGTGGAGGTTCA<br>rev: AAGGTTGGGCCACCAAGAAC
nt control-sgRNA	fwd: GCGAGGTATTCGGCTCCGCG<br>rev: TAATGCCAACTTTGTACAAGAAAG
Cartpt-sgRNA	fwd: ACCGGACGTGGTACCAGTCC<br>rev: TAATGCCAACTTTGTACAAGAAAG
